# Preliminary assessment of the therapeutic potential of staphylococcal enterotoxin-like W via biological activity and TCR binding sites analysis

**DOI:** 10.1080/21505594.2025.2550622

**Published:** 2025-08-31

**Authors:** Yuhua Yang, Xiaoyue Wei, Fanliang Meng, Yanan Gong, Yahui Guo, Lijin Long, Jiaming Fan, Yakun Zhao, Wanting Wang, Di Xiao, Lei Wang, Maojun Zhang, Dongliang Hu, Jianzhong Zhang, Xiaomei Yan

**Affiliations:** a National Key Laboratory of Intelligent Tracking and Forecasting for Infectious Diseases, National Institute for Communicable Disease Control and Prevention, Chinese Center for Disease Control and Prevention, Beijing, China; b Infectious Disease Prevention and Control Department, Bayannur Center for Disease Control and Prevention, Inner Mongolia Autonomous Region, China; c School of Public Health, China Medical University, Shenyang, China; d Department of Zoonoses, Kitasato University School of Veterinary Medicine, Towada, Japan

**Keywords:** Superantigen, staphylococcal enterotoxin-like W, sequence polymorphism, stability, site-directed mutagenesis, antitumor

## Abstract

Staphylococcal enterotoxin-like W (SElW) is a novel, widely prevalent enterotoxin-like protein that functions as a classical staphylococcal superantigen (SAg) and has been shown to exacerbate infections caused by the *S. aureus* epidemic clone CC398. However, the genetic distribution and amino acid polymorphisms, biological and antitumor activity, and T cell receptor (TCR) binding sites of SElW in *S. aureus* strains prevalent in China have not been investigated. The carrier rate and distribution of *selw* were determined by PCR, the stability and antitumor activity of recombinant SElW (rSElW) protein were evaluated. The superantigen activity of the five mutants (Y18A, N19A, W55A, C88A, and C98A) was compared to that of wild-type SElW (WT-rSElW) to assess the role of these sites in mediating TCR binding. The *selw* gene was detected in all (986/986, 100%) dominant clonal lineages of *S. aureus* and most strains (69.1%, 56/81) had a full-length *selw* open reading frame with a sequence identity of 90.5%. rSElW was heat-stable but not resistant to pepsin and trypsin digestion. Additionally, rSElW significantly inhibited the proliferation of MCF-7 and AGS, but not A549 *in vitro*. The rSElW mutants C88A and C98A markedly reduced T cell proliferation and IL-2, IFN-γ and TNF-α secretion compared to WT-rSElW. rSElW is a highly prevalent SAg that binds to the TCR via C98 and C88, which may serve as novel therapeutic targets for *S. aureus* infections and its application in anti-tumor activity needs to be further evaluated *in vivo.*

## Introduction

*Staphylococcus aureus* is an opportunistic pathogenic bacterium that colonizes the nasal vestibular mucosa and various skin surfaces in humans [1–3] and can cause skin and soft tissue infections, endocarditis, and bacteremia [4–6]. *S. aureus* infects the host and secretes a wide range of virulence factors, including hemolysins, exfoliative toxins, and staphylococcal superantigens (SAgs) [7,8]. SAgs, including staphylococcal enterotoxins (SEs), staphylococcal enterotoxin-like toxins (SEls), and toxic shock syndrome toxin-1 [9,10], are potent T cell mitogens that bind directly to major histocompatibility complex class II (MHC-II) molecules on antigen-presenting cells and form bridges with the variable regions of the T cell receptor (TCR) α- or β-chain without undergoing antigen processing and presentation [11,12]. This interaction induces abnormally rapid proliferation of T cells and massive release of pro-inflammatory cytokines such as interleukin-2 (IL-2), IL-4, tumor necrosis factor (TNF), and interferon-γ (IFN-γ) [13–15].

At least 29 enterotoxins with superantigenic and emetic properties have been reported. Enterotoxins demonstrated to induce emesis in mammals are designated as “SEs,” which include SEA-SEE [16], SEG-SEI [17,18], SEK-SEQ [19,20], SER-SET [21,22], SEY [23], SE01, and SE02 [24]. In contrast, those without emetic properties or not evaluated in non-human primate models of emesis are designated as SEls, such as SElJ, SElU-SElX, SElZ, SEl26, and SEl27 [25–27]. SEs and SEls are structurally similar in that they are non-glycosylated single-chain homologous globular proteins with distinct antigenicity and low molecular weight (19–29 kDa) [28].

SEs not only activate T cells to secrete cytokines that inhibit tumor growth but also stimulate cytotoxic T cells to kill MHC II-positive tumors under low-dose conditions, making them effective antitumor agents [29]. In China, staphylococcal enterotoxin C2 (SEC2) injection has been used in cancer treatment since 1999 [30,31]. Additionally, SEs have been shown to act synergistically with antibodies to recruit T cells [32]. However, the toxic side effects such as the emetic activity and strong superantigenic reaction of enterotoxin itself have limited its clinical application. The identification of enterotoxins exhibiting low cytotoxic potential while maintaining potent antitumor efficacy represents a scientifically valuable research direction in oncotherapeutic discovery.

Identifying the TCR binding sites for SEs is crucial for gaining insights into their structural and functional activities, as well as their mechanisms of immune recognition, which will aid in developing SAgs antagonists and enhancing their anticancer potency. Protein structure prediction and site-directed mutagenesis are two common techniques for studying TCR binding sites. Xu et al. revealed that the TCR binding sites on SAgs are located within a shallow groove between two domains of the staphylococcal SAgs [10]. These binding sites are characterized by several cross-interface bonds, with N23 of SEB and SEC2, or N16 of SEH, identified as the key residue mediating TCR binding. SElW is a newly identified enterotoxin-like toxin that shares 36% homology with SEA [33]. Extensive genomic data analysis showed that the *selw* is present in over 90% of *S. aureus* isolates and in almost all dominant clonal lineages of *S. aureus* [13,34–36]. Research has shown that SElW exhibits superantigenicity and induces massive T cell proliferation and IL-2 secretion. Animal models of bacteremia have demonstrated that SElW can increase the bacterial load in the liver [13]. Furthermore, SElW is the only SAg predicted to be present in the epidemic clone CC398, and its deletion in CC398 abrogated its capacity to promote T cell proliferation *in vitro*, suggesting that this protein may be associated with immune recognition [13,37,38].

In this study, we analyzed the genetic distribution, amino acid polymorphisms, biological and antitumor activity, and TCR binding sites of SElW in *S. aureus* strains prevalent in China to gain further insights into its antitumor properties and therapeutic potential.

## Material and methods

### Bacterial strains and culture conditions

A total of 986 *S. aureus* strains, with 831 from 16 provinces of China across 9 distinct food substrates and obtained from 6 provinces representing various hosts including healthy individuals, clinical patients, and porcine sources. These isolates exhibited broad genetic diversity, encompassing multiple sequence type (ST) profiles, *spa* polymorphisms, and clonal cluster variations. All strains of *S. aureus* are stored in our laboratory. All *S. aureus* strains were cultured on Colombia blood agar plates at 37°C for 14–16 h. *E. coli* DH5α (Invitrogen, USA) was used for cloning and BL21(DE3) (Novagen) was used as the expression host. Competent *E. coli* was cultured aerobically at 37°C in Luria-broth (LB) medium containing kanamycin (25 μg/mL).

### Plasmid and cell lines

The cloning vector pMD18-T was purchased from Takara Biotechnology (Dalian, China), and the expression vector pET-28a (+) was stored in our laboratory. Human gastric cancer cell line AGS, lung adenocarcinoma cancer cell line A549, and breast cancer cell line MCF-7 were obtained from the American Type Culture Collection (ATCC). Human peripheral blood mononuclear cells (PBMCs) were purchased from Saily Biotechnology Co. (Shanghai, China).

### Sequence analyses

A whole-genome sequence dataset of food-derived *S. aureus* strains (*n* = 831) from China was used to examine the genetic distribution of *selw* [39,40]. The *selw* gene in these strains was identified using nucleotide BLAST (blastn) [41] with ≥80% coverage and sequence identity. In addition, the *selw* was detected in 155 strains from patients (*n* = 124), healthy individuals (*n* = 28), and pigs (*n* = 3) in China by PCR and Sanger sequencing. The sequencing primers were synthesized by Sangon Biotech (Shanghai, China) and listed in Supplementary Table S1. Multiple sequences alignment was performed across 81 strains belonging to 54 sequence types (ST; 19 clonal complexes). A phylogenetic tree was constructed using maximum likelihood estimation with 1000 bootstrap iterations and visualized by MEGA X and iTOL (https://itol.embl.de) [42].

### Reverse-transcription PCR (RT-PCR)

Total RNA was extracted from 13 *S. aureus* strains of different STs and origins (Supplementary Table S2) using RNAiso plus (Takara Biotechnology Corp., Dalian, China) and 1 μg of RNA was reverse transcribed into cDNA using the Primescript RT Master Kit (Takara Biotechnology Corp., Dalian, China). The selw gene was amplified by PCR using the primers *selw*-Fw and *selw*-Rw (Supplementary Table S1). The PCR reaction conditions were as follows: 94°C for 2 min, 35 cycles of 98°C for 10 s, 63°C for 30 s and 68°C for 30 s, followed by 72°C for 10 min.

### Cloning, expression, and purification of recombinant SElW (rSElW)

The *selw* gene of the *S. aureus* strain DC51619 was cloned into the expression vector pET-28a (His-tag) and amplified by the primers rSElW-F and rSElW-R (Supplementary Table S1). rSElW proteins with a non-cleavable N-terminal 6×His-tag were produced in *E. coli* according to previous methods [43]. *E. coli* BL21(DE3) containing the pET-28a-*selw* plasmid was cultured in LB with kanamycin and induced with a final concentration of 1 mM isopropyl-β-D-thiogalactopyranoside (IPTG) (TransGen Biotech, Beijing, China) for 12 h at 16°C during mid-exponential phase (OD_600_ = 0.6–0.8). Proteins were purified by affinity chromatography (AKTA fast protein liquid chromatography (FPLC) system explorer 100) on a HisTrap column (GE healthcare, Buckinghamshire, UK). Then, the protein content in the suspension was determined by the Pierce™ BCA Protein Assay (Thermo Fisher Scientific, US), while endotoxin levels were evaluated using the Pierce™ Chromogenic Endotoxin Quant Kit (Thermo Fisher Scientific, US), in strict accordance with the manufacturers’ guidelines. Finally, protein purity was verified by sodium dodecyl sulfate – polyacrylamide gel electrophoresis (SDS-PAGE).

### Western blot identification of SElW

Monoclonal and polyclonal antibodies (Biodragon lmmunotechnologies Co., Ltd., Beijing, China) were obtained by immunizing mice and rabbits with rSElW, respectively. Both prepared antibodies were stored in our laboratory. Eight *S. aureus* strains were cultured in brain heart infusion broth with 200 rpm shaking at 37°C for 14–16 h. Culture supernatants were harvested by centrifugation, concentrated to 1/10th of the original volume using Amicon-Ultra-15 Centrifugal Filter units (10,000 Da MWCO) (Millipore, Merck, Germany), passed through 0.22 μm filters, and stored at −20°C. Bacterial proteins were extracted from eight *S. aureus* isolates by cell lysis, and 20 μg of lysate of each strain was separated by SDS-PAGE and transferred onto a 0.22 μm PVDF membrane (Millipore, MA, USA). The membrane was blocked with 5% skim milk powder in PBS for 2 h at room temperature, incubated with primary monoclonal antibody in blocking buffer at 4°C overnight, washed 3 times with TBST, and incubated with goat anti-mouse IgG-HRP (ZSGB-Bio, Beijing, China) for 1 h at room temperature. After washing, the membrane was incubated with ECL substrate (Takara Biotechnology Dalian, China) and the protein bands were visualized using an ultrasensitive quantitative fluorescent gel imaging system (Amersham lmager 680, GE healthcare). Information on the test strains is detailed in Supplementary Table S2. The same procedures were followed for the detection of SElW using primary rabbit polyclonal antibody and goat anti-rabbit secondary IgG-HRP (ZSGB-Bio, Beijing, China).

### HPLC-MS/MS identification of SElW

Bacterial proteins were extracted from the DC50005 and DC51908 strains and separated by SDS-PAGE. Protein bands with a molecular weight of 25–35 kDa were cut out from the gel and digested as described previously using rSElW as a reference [44]. After digestion, the digested proteins were passed through a MonoSpin® C18 desalination column according to the manufacturer’s instructions and dried by vacuum at 4°C. The protein samples were analyzed using HPLC-MS/MS as described previously [44] and the resulting protein data were processed with Proteome Discoverer (version 1.4).

### Stability assay of rSElW

The stability of rSElW was evaluated as described previously [45–47] using BSA (Sigma, Germany) and rSEA (produced in our lab) as controls. To test the heat stability of rSElW, 500 μL of 100 μg/mL rSElW in PBS was heated in a heat block at 100°C for 0, 0.5, 1, 2, 4, 6, 8, or 10 h.

BSA, rSEA and rSElW were digested by pepsin (Sigma-Aldrich, Germany) to assess the proteolytic stability of rSElW. Each protein (final concentration 100 μg/mL) was incubated with pepsin (100 μg/mL in 0.1 M sodium acetate buffer, pH 4.5) in a final volume of 500 μL at 37°C for 0, 0.5, 1, 2, or 4 h [45].

Similarly, each protein (final concentration 100 μg/mL) was incubated with trypsin (Sigma-Aldrich, Germany; 50 μg/mL in 0.01 M Tris-HCl, pH 8.0) in a final volume of 500 μL at 37°C for 0, 0.5, 1, 2, 4, 6, or 8 h. The pepsin and trypsin digestion reactions were terminated by heating the samples at 95°C for 5 min, followed by immediate cooling before loading for SDS-PAGE. After electrophoresis, the gel was stained with Coomassie Brilliant Blue for protein band visualization.

### *In vitro* antitumor activity assay

The antitumor activity of rSElW was evaluated using human PBMCs as effector cells and MCF-7 breast cancer cells, AGS gastric cancer cells, and A549 lung cancer cells as target cells. Tumor cells (5 × 10^3^ cells/100 µL) and human PBMCs (8 × 10^4^ cells/100 µL) were seeded in separate 96-well flat-bottom plates and cultured in RPMI 1640 medium (Gibco, UK) with 10% (v/v) heat-inactivated fetal bovine serum (Gibco, UK), 100 U/mL penicillin, and 100 μg/mL streptomycin at 37°C with 5% CO_2_ for 24 h. Human PBMCs were treated with a final concentration of 10^0^-10^−7^ µg/mL rSElW in a total volume of 280 μL/well and cultured at 37°C for another 24 h. Discarded the supernatant of tumor cells, the cultured human PBMCs and rSElW mixtures were added at 280 μL/well into the tumor cell plates at a 16:1 effector: target ratio and incubated at 37°C for 72 h. Human PBMCs, tumor cells, RPMI 1640 medium, and BSA were used as negative controls. After incubation, the cells were washed three times with RPMI 1640 medium and treated with 100 µL/well of RPMI 1640 containing 10 µL of CCK-8 solution (Beyotime Biotechnology, Shanghai, China) for 2 h, followed by the measurement of optical density (OD) at 450 nm using a microplate reader (Thermo Scientific, USA). Each experiment was repeated three times, and each sample was tested in triplicate wells. The half maximal inhibitory concentration (IC50) was determined by nonlinear regression analysis (curve fit) of the concentration – response curve. Tumor growth inhibition (TGI) rate was calculated by:

TGI rate = 100 - [(OD of experimental group – OD of human PBMCs)/(OD of tumor cell control group – OD of blank group)] × 100%.

### Cloning, expression, and purification of mutant SElW-Y18A, SElW-N19A, SElW-W55A, SElW-C88A, and SElW-C98A

In our previous study, the simulation of SElW docking on the TCR revealed five important binding sites, namely Y18, N19, W55, C88, and C98 [43]. To verify the role of these residues in mediating TCR binding, alanine mutations were introduced into the pET-28a-*selw* vector by site-directed mutagenesis and mutant SElW were constructed by overlap extension PCR using the chromosomal DNA of *S. aureus* DC51619 as a template. All primers used are listed in Supplementary Table S1. The PCR reaction conditions were as follows: 1 cycle of 98°C for 30 s, 35 cycles of 98°C for 8 s, 55°C for 20 s and 72°C for 25 s, followed by 72°C for 10 min. The mutant proteins were confirmed by DNA sequencing and subcloned into the pET-28a expression vector. All mutant proteins were expressed in *E. coli* BL21 (DE3) cells and purified by His-Trap FF crude nickel affinity column followed by ion exchange chromatography. Protein purity was verified by SDS-PAGE. Following endotoxin removal and protein concentration determination, the samples were stored at −80°C.

### T cell proliferation assays of wild-type and mutant SElW

Human PBMCs were seeded in 96-well flat-bottom plates at 1x10^6^ cells/mL in RPMI 1640 medium containing 10% (v/v) heat-inactivated fetal bovine serum, 100 U/mL penicillin, and 100 μg/mL streptomycin, and then treated with 10-fold dilutions of wild-type (WT) or mutant proteins (1 to 10^−5^ μg/mL) for 72 h at 37°C with 5% CO_2_. Human PBMCs treated with rSEA (1 to 10^−5^ μg/mL) were used as the positive control, while those treated with or without 1 μg/mL of BSA were used as the negative and blank controls, respectively. After incubation, the supernatant was removed and the cells were treated with fresh medium containing 10% CCK-8 reagent (v/v) (Beyotime Biotechnology, Shanghai, China) at 37°C for 2 h. OD_450_ was measured to calculate the proliferation index (PI): PI = (OD of experimental wells – OD of blank)/(OD of negative control wells – OD of blank). The experiments were independently conducted three times, with at least three biological replicates.

### Cytokine assay of WT and mutant SElW

Human PBMCs were stimulated with rSEA and WT-rSElW and mutant rSElW as described above. Cell culture supernatants were collected for the detection of IL-2, IFN-γ, and TNF-α using ELISA kits (Invitrogen, USA) according to the manufacturer’s instructions. OD_450_ was measured using a microplate reader. Each sample was tested in triplicate wells and each experiment was repeated three times.

### Statistical analysis

Data were statistically analyzed using one-way ANOVA in Origin 2017 (USA) and graphs were generated using Adobe Illustrator CS5 (USA). Data are expressed as mean ± standard deviation (mean ± SD). *p* < 0.05 was considered as statistically significant.

## Results

### Genetic distribution and sequence polymorphisms of SElW

The *selw* gene was identified in all 986 (100%) *S. aureus* strains examined and was prevalent among the endemic clonal lineages CC1, CC5, CC6, CC7, CC8, CC9, CC22, CC25, CC45, CC59, CC72, CC121, and CC398.

**Figure 1.**
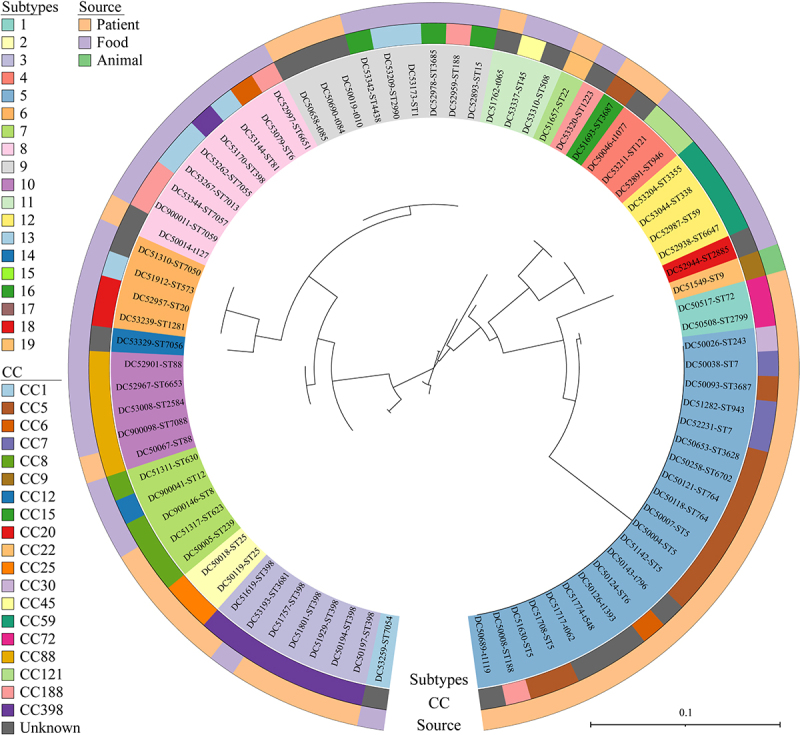
Phylogenetic analysis of SElW amino acid sequences by maximum-likelihood estimation. The tree was generated using the iTOL online platform (https://itol.embl.de/). The outer, middle, and inner rings represent the source, CC, and subtypes of SElW, respectively. CC, clonal complexes.

Sequence comparisons of 81 strains from 54 STs (19 clonal complexes) showed a less conserved N-terminus compared to the C-terminus, with a 90.5% amino acid sequence identity (Supplementary Figure S1). Phylogenetic analysis of the SElW amino acid sequences revealed 19 distinct subtypes, including subtype 5 (25.9%, 21/81), subtype 8 (11.1%, 9/81), subtype 9 (11.1%, 9/81), subtype 3 (8.6%, 7/81), subtype 7 (6.2%, 5/81), and subtype 10 (6.2%, 5/81). Full-length, intact *selw* variants were identified in 56 (69.1%) strains across 14 clonal complexes, while truncated *selw* caused by premature stop codons at base 110 or 139 were detected in the remaining 25 (30.9%) strains across 7 clonal complexes, with most belonging to subtype 5 (25.9%, 21/81), followed by subtype 13 (2.5%, 2/81), subtype 19 (1.2%, 1/81), and subtype 16 (1.2%, 1/81). Intact and truncated *selw* alleles were present in both human and animal *S. aureus* strains. Additionally, truncated *selw* alleles were predominantly found in clones CC5, CC6, CC7, CC9, CC30, CC72, and CC188 (Figure 1).

### SElW transcript and protein expression

*selw* transcripts (241 bp) were amplified by RT-PCR in 13 strains from 6 clonal complexes (Supplementary Figure S2A). HPLC-MS/MS analysis of SElW proteins in 2 of the 13 strains revealed 9 peptide matches (45% coverage) between strain DC51908 and rSElW, and 6 peptide matches between strain DC50005 and rSElW (32% coverage) (Table 1).

**Table 1.** Identification of SElW from *S. aureus* strains by HPLC-MS/MS.

Accession	Description	Length of protein (AAs)	MW [kDa]	Cal. pI	Coverage [%]	Peptides	Unique Peptides	PSMs	Score Sequest HT: Sequest HT	Peptides (by Search Engine): Sequest HT
rSElW	SElW	222	26.1	6.29	55	20	20	188	536.48	20
DC51908	SElW	222	26.1	6.29	45	9	9	14	45.66	9
DC50005	SElW	222	26.1	6.29	32	6	6	8	23.03	6

Abbreviations: AA, amino acids; Cal. pI, calculated isoelectric point; MW, molecular weight; PSM, peptide spectrum match.

**Figure 2.**
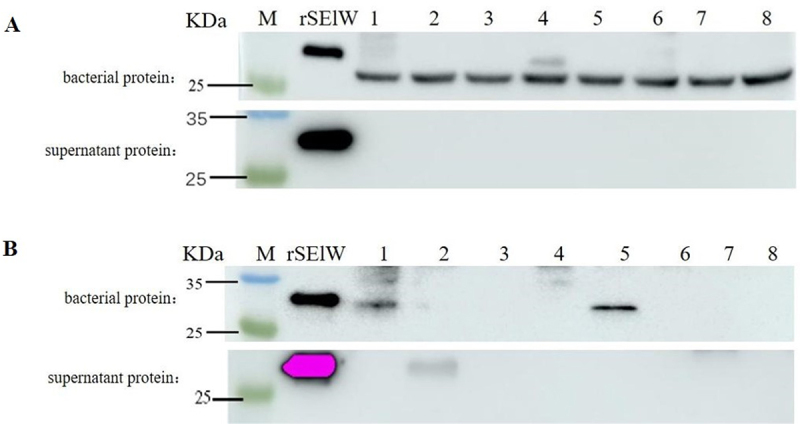
SElW protein expression in *S. aureus* strains detected by WB. (A) SElW protein detection using mouse monoclonal antibody; (B) SElW protein detection using rabbit polyclonal antibody. Numbers 1–8: different *S. aureus* strains; rSElW: positive control; M: Marker.

SElW expression in eight *S. aureus* isolates was also detected by Western blot (WB) using rabbit polyclonal and mouse monoclonal antibodies, which have been confirmed to specifically react with rSElW but not rSEA (Supplementary Figure S2B, C). WB with the mouse monoclonal antibody showed that SElW was present in the bacterial proteins but absent from the culture supernatant of the eight isolates (Figure 2(A)). Furthermore, WB with the rabbit polyclonal antibody detected SElW in the bacterial proteins of two isolates and in the culture supernatant of one isolate (Figure 2(B)).

### Stability of rSElW

**Figure 3.**
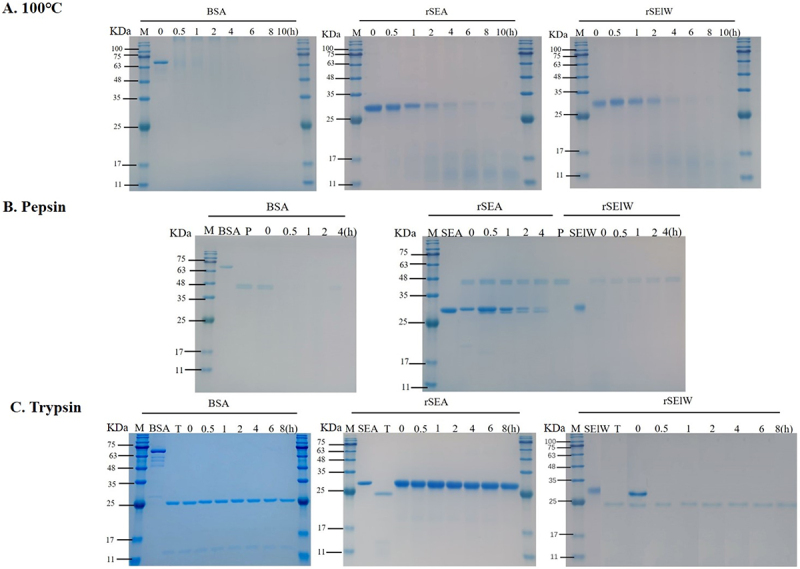
Heat and proteolytic stability of rSElW. (A) Heat stability of rSElW. 100 μg/mL of BSA, rSEA, and rSElW were heated at 100°C for the indicated time and analyzed by SDS-PAGE. (B) Pepsin stability of rSElW. 100 μg/mL of BSA, rSEA, and rSElW were treated with pepsin (100 μg/mL) at 37°C for the indicated time at pH 4.5. (C) Trypsin stability of rSElW. 100 μg/mL of BSA, rSEA, and rSElW were treated with trypsin (50 μg/mL) at 37°C for the indicated time at pH 8.0. M: Marker. P: Pepsin; t: trypsin.

The heat stability test showed that rSElW was degraded after heating at 100°C for 4 h, whereas rSEA was completely degraded after 8 h, and the control BSA was fully degraded after just 1 h (Figure 3(A)). The digestion tolerance test with pepsin demonstrated that both rSElW and BSA were degraded within 0.5 h, whereas rSEA remained stable for 4 h (Figure 3(B)). Similarly, rSElW and BSA were degraded by trypsin after 0.5 h, while rSEA remained intact at 8 h post-digestion (Figure 3(C)).

### *In vitro* antitumor activity of rSElW

**Figure 4.**
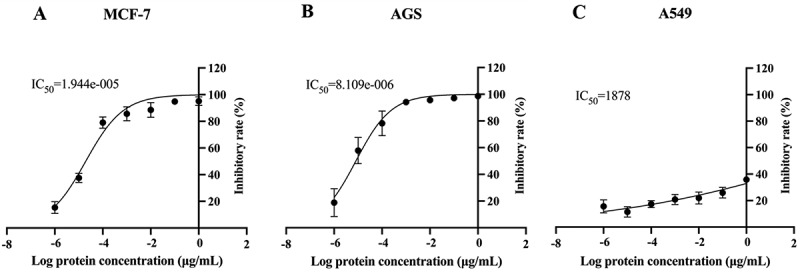
Inhibition of tumor cell growth by rSElW *in vitro*. PBMCs stimulated with 10^0^ to 10^−6^ μg/mL of rSElW were co-cultured with (A) MCF-7, (B) AGS, or (C) A549 cells at an effector to target cell ratio of 16:1 for 72 h. BSA and untreated cells were used as negative controls. The data represent the mean ± SD of three independent experiments. *p < 0.05.

The inhibitory effects of rSElW-activated PBMCs on MCF-7, AGS, and A549 cell proliferation were examined using the CCK-8 assay. As shown in Figure 4 and Supplementary Figure S3, rSElW simulation significantly inhibited MCF-7 and AGS cell proliferation *in vitro* (*p* < 0.05) compared with the negative control. The rate of PBMC-mediated MCF-7 and AGS cell inhibition increased in a dose-dependent manner as rSElW concentration increased, achieving 80% TGI rate at a concentration of 10^−4^ μg/mL and IC_50_ of 1.9 × 10^−5^ μg/mL and 8.1 × 10^−5^ μg/mL, respectively. On the other hand, rSElW marginally inhibited A549 cell growth (IC_50_ of 1878 μg/mL) compared to those observed in MCF-7 and AGS cells.

## Superantigenic activity of rSElW mutants

**Figure 5.**
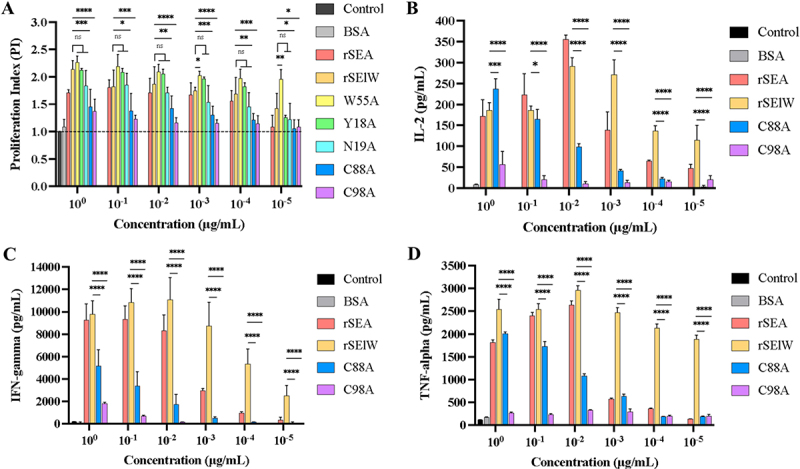
Proliferative capacity of human PBMCs and their cytokine secretion profiles after stimulation with different concentrations of rSEA, rSElW, and Y18A, N19A, W55A, C88A, C98A mutants. (A) PBMCs were treated with rSEA, rSElW or mutant proteins for 72 h; (B, C, D) ELISA detection of IL-2, IFN-γ and TNF-α levels from PBMCs stimulated with different concentrations of rSEA, rSElW, C88A, and C98A. Untreated PBMCs and PBMCs treated with BSA were used as negative controls. Data represent the mean ± SD of three independent experiments. *p < 0.05, **p < 0.01, ***p < 0.001, ****p < 0.0001, NS: not significant.

The SElW mutants Y18A, N19A, W55A, C88A, and C98A were cloned into pET-28a vectors and subsequently expressed and purified from *E. coli* BL21(D3) (Supplementary Figure S4A-C). The superantigenic activities of these mutants were assessed using PBMC proliferation and cytokine production assays. First, the mitogenic effect of the five rSElW mutants on human PBMCs was evaluated by the CCK-8 assay using rSElW and rSEA as controls (Figure 5(A)). Notably, the C88A and C98A mutants showed significantly lower mitogenic activity on human PBMCs at the concentration of 10^0^−10^−4^ μg/mL compared with WT-rSElW (*p* < 0.001). The proliferative capacity of PBMCs stimulated by N19A was also reduced, but the difference was not statistically significant. In contrast, Y18A and W55A exhibited marginally higher mitogenic activities on human PBMCs compared with rSElW (*p* > 0.05).

Next, we further examined IL-2, IFN-γ and TNF-α production from PBMCs treated with different concentrations of rSEA, rSElW and the C88A and C98A mutants. Similar to the findings from the proliferation assay, IL-2, IFN-γ and TNF-α productions were significantly lower from C98A-stimulated PBMCs compared to rSElW-stimulated PBMCs at 10^0^-10^−5^ μg/mL (*p* < 0.0001). On the other hand, C88A significantly downregulated IFN-γ and TNF-α expression at 10^0^-10^−5^μg/mL and inhibited IL-2 secretion at 10^−1^-10^−5^μg/mL but not at 10^0^ μg/mL. Taken together, these data suggested that the C98 and C88 residues are critical for TCR binding by SElW (Figure 5(B–D)).

## Discussion

Our study demonstrated that the *selw* gene is present in all 986 tested *S. aureus* strains from various sources in China and is widely distributed across 13 endemic clonal lineages, which is in agreement with findings reported in other countries [13,34,48]. Additionally, sequence comparisons revealed that polymorphisms in SElW were predominantly located in the N-terminus of the protein. Vrieling et al. demonstrated that different SElW allelic variants exhibit distinct superantigenic activity and Vβ T cell activation, independent of the *S. aureus* host species. Notably, two SElW variants were found to strongly activate human Vβ21 T cells [13]. Whether this finding applies to the dominant SElW variants in different clone complexes in China requires further investigation. Additionally, our data indicated a higher percentage of *S. aureus* strains harboring a full-length, intact *selw* gene variant compared to that reported by Vrieling et al. (69.1% vs. 62.5%) [13], which may be attributed to differences in sample selection.

We observed that several dominant *S. aureus* strains in China transcribed and expressed the *selw* gene. However, SElW protein levels were notably low in the culture supernatant, potentially due to either minimal protein expression at the time of collection or the lack of protein secretion under the current culture conditions. The presence of SE genes on various genetic elements contributes to the complexity and diversity of their expression. Several studies demonstrated that the elements Agr, σB, Rot, several Sar homologs, SaeR, and phage life cycle played a role in the regulation of SE expression [49–52]. Furthermore, environmental conditions such as temperature, food types, salt content, water activity, pH, and competing strains further modulated SE expression [50,53]. SElW is a core genome-encoded SAg [13,33], but its regulatory mechanism remains unclear. Thus, further studies are warranted to elucidate the regulatory mechanisms and the various environmental conditions affecting SElW expression.

Several SEs have been shown to be resistant to heat and digestive enzymes, contributing to food contamination and gastrointestinal infections [24,54,55]. Our results demonstrated that SElW exhibited heat resistance comparable to that of SEA, indicating that SElW can withstand degradation by conventional cooking methods. However, SElW was susceptible to pepsin and trypsin digestion, implying that the protein is degraded upon entry into the gastrointestinal tract and therefore unlikely to induce emesis.

As a member of the SE family, SEC2 has been extensively used as a clinical tumor immunotherapeutic agent in China for over two decades [56]. However, the widespread application of enterotoxins has been constrained by their toxic side effects, which can be addressed by utilizing enterotoxin-like proteins with minimal or absent emetic activity, making them promising candidates for clinical drug development [14]. In this study, we found that rSElW strongly inhibited MCF-7 and AGS cell proliferation, achieving a TGI rate of 80% at a concentration of 10^−4^ μg/mL, which is comparable to those of other enterotoxins, such as SEA, SEB, and SEQ [20,57–60]. The antitumor activities of SEs are predominantly driven by cell-mediated immunity, particularly through immune responses induced by cytotoxic T cells [61,62]. In tumor tissues, CD8^+^ T cells are activated through TCR recognition of tumor antigens presented by MHC class I molecules on tumor cells [63]. Moreover, SEs mutants have been shown to enhance the tumor immune microenvironment, suggesting their potential as a strategy to boost immune responses. Notably, SElW lacks the emetic activity that is characteristic of other enterotoxins (unpublished data). This unique property, combined with its observed antitumor potential, underscores its promise for both mechanistic research and clinical applications. Therefore, characterizing the *in vivo* antitumor mechanisms and activities of rSElW and SElW mutants will be the focus of future research.

In our previous work, molecular docking simulations of SElW with the TCR revealed five putative TCR binding sites, namely Y18, N19, W55, C88, and C98 [43]. To evaluate the role of these sites in mediating TCR binding, SElW Y18A, N19A, W55A, C88A, and C98A mutants were constructed by site-directed mutagenesis. It has been shown that SEB and SEA interact with TCRV_β_ through distinct residues within the shallow groove between the β-barrel and α_2_-helix at the N-terminus [64,65]. Rödström et al. identified a common hot spot asparagine residue within the α_2_-helix of five SEs (SEA N25, SEB N23, SEC N23, SEE N21, and SEH N16) that played a significant role in SAg-TCR complex formation and binding energy [65]. In contrast, the same residue (N19) in SElW had no significant impact on SElW-TCR complex formation (*p* > 0.05). Our result showed that C98 and C88 were the key residues affecting the superantigenic activity of SElW, as mutations at both residues significantly attenuated PBMC proliferation and IL-2, IFN-γ and TNF-α production. SElW C98/C88, which corresponds to SEA C106/C96, SEB C113/C93, SEC2 C110/C93, SED C106/C96, and SEE C106/C96, are conserved sites in the SEs sequence found within the disulfide loop of the β-grasp domain at the C-terminus of SElW [66–70]. This disulfide loop is a highly conserved structural feature among all SEs and is critical for the stability, structure, and biological functions of enterotoxins [71–74]. Mutations at SEC2 C110/C93 and SEA C106/C96 abolished their ability to promoter murine T cell proliferation and cytotoxicity *in vitro*, suggesting that the cysteine residues in the disulfide loop are essential for the superantigenic functions of SEC2 and SEA [70,75]. Similarly, we showed for the first time that the C98/C88 residues in the β-grasp disulfide loop are critical for the superantigenicity of SElW. Additionally, the SElW C98A mutant resulted in significantly greater inhibition of cytokine production compared to the C88A mutant, suggesting that the superantigenic activity of SElW is predominantly contributed by C98. This contrasts with the finding on SEC2, where C93, rather than C110, plays a more critical role in its superantigenic activity [70]. This inconsistency may be attributed to subtle differences in protein structures and proximity to the MHC II binding sites. Since C93 is closer to both the TCR and MHC II binding sites in SEC2, a mutation at C93 could lead to a more pronounced impairment of T cell response than a mutation at C110 [76,77]. Furthermore, it would be worthwhile to investigate whether simultaneous mutations at both the C88 and C98 sites could further diminish the binding affinity between SElW and TCR, potentially impacting the synergistic effect on T cell mitotic activity.

In conclusion, we found that the *selw* gene is broadly distributed among the endemic clonal lineages of *S. aureus*. SElW is resistant to heat but susceptible to enzymatic digestion. Our study is the first to demonstrate that the C98 and C88 residues in the disulfide loop of the β-grasp domain are essential for the superantigenic functions of SElW. Altogether, these findings offer valuable insights to the pathogenesis of *S. aureus* and highlight its potential as a novel therapeutic target for *S. aureus* infections. Additionally, the antitumor activity of SElW observed *in vitro* underscore its promise for both mechanistic studies and clinical research.

## Supplementary Material

Supplementary Figure S3_Revised.tif

Supplementary Table S1.docx

Clean copy of Supplementary Table S2- QVIR-2025-0062.R1.docx

Supplementary Figure S4.tif

Supplementary Figure S1.tif

Supplementary Figure S2.tif

## Data Availability

The data that support the findings of this study are openly available in Figshare at https://doi.org/10.6084/m9.figshare.28440671.v1.
