# Supplementary figures and images for: Preliminary assessment of the therapeutic potential of staphylococcal enterotoxin-like W via biological activity and TCR binding sites analysis
Source: Virulence. 2025 Aug 31;16(1):2550622. doi: 10.1080/21505594.2025.2550622 (PMC12407819; doi:10.1080/21505594.2025.2550622)

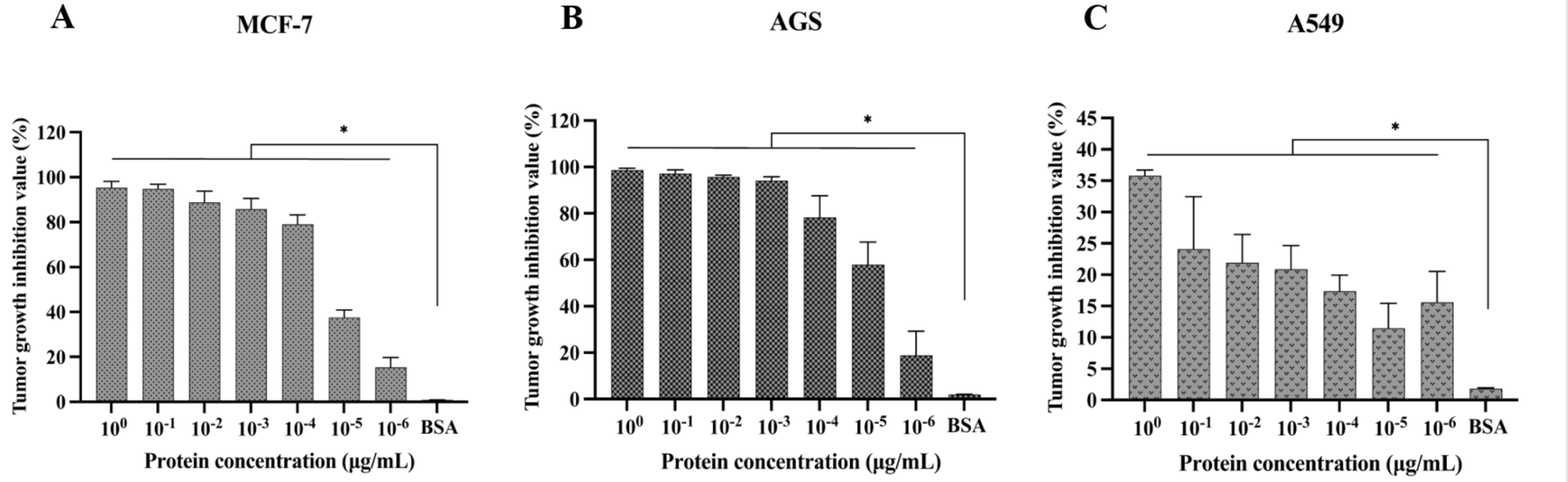

Supplement: Supplementary Figure S3_Revised.tif [file KVIR_A_2550622_SM9389.tif]

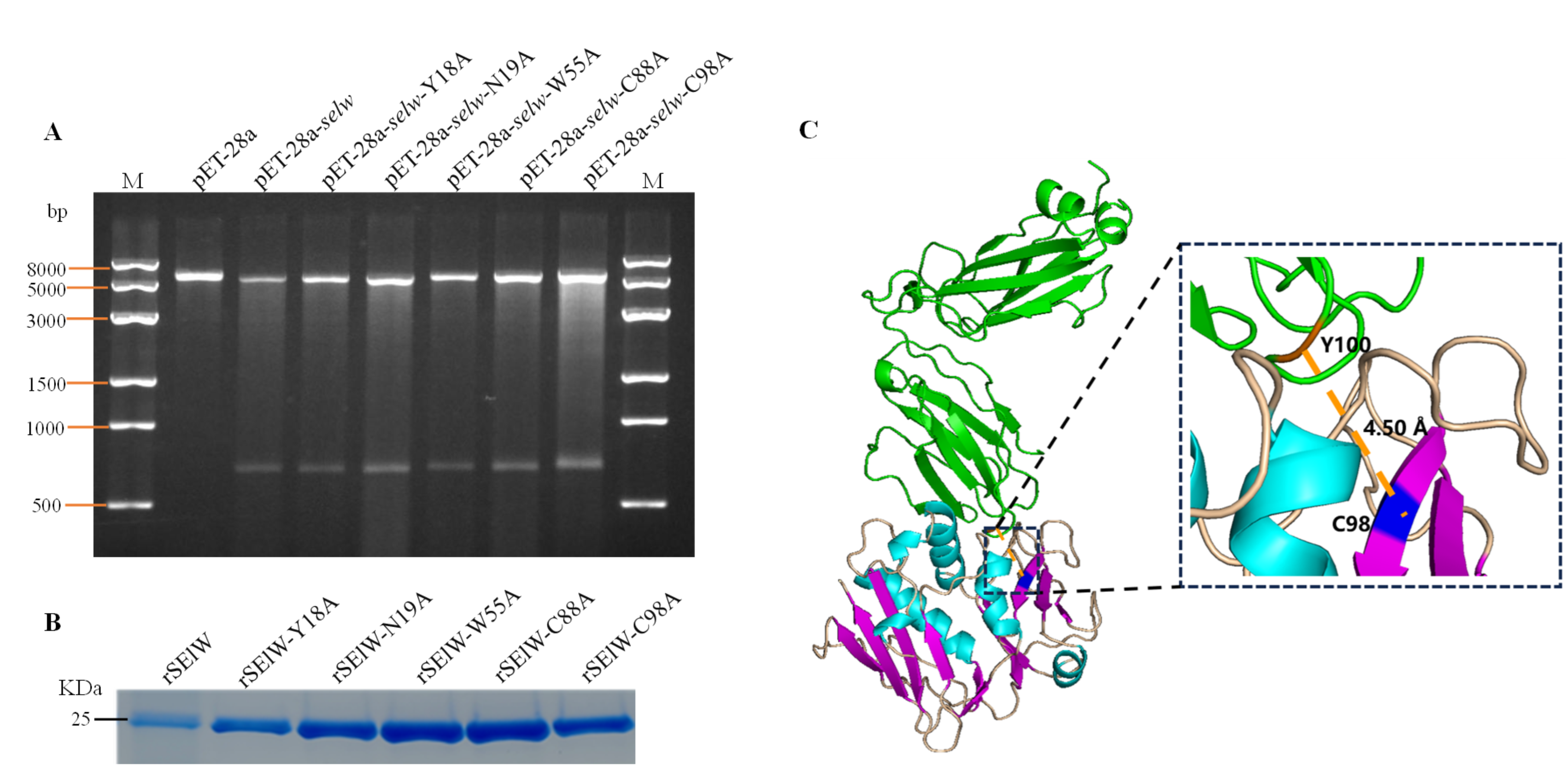

Supplement: Supplementary Figure S4.tif [file KVIR_A_2550622_SM9386.tif]

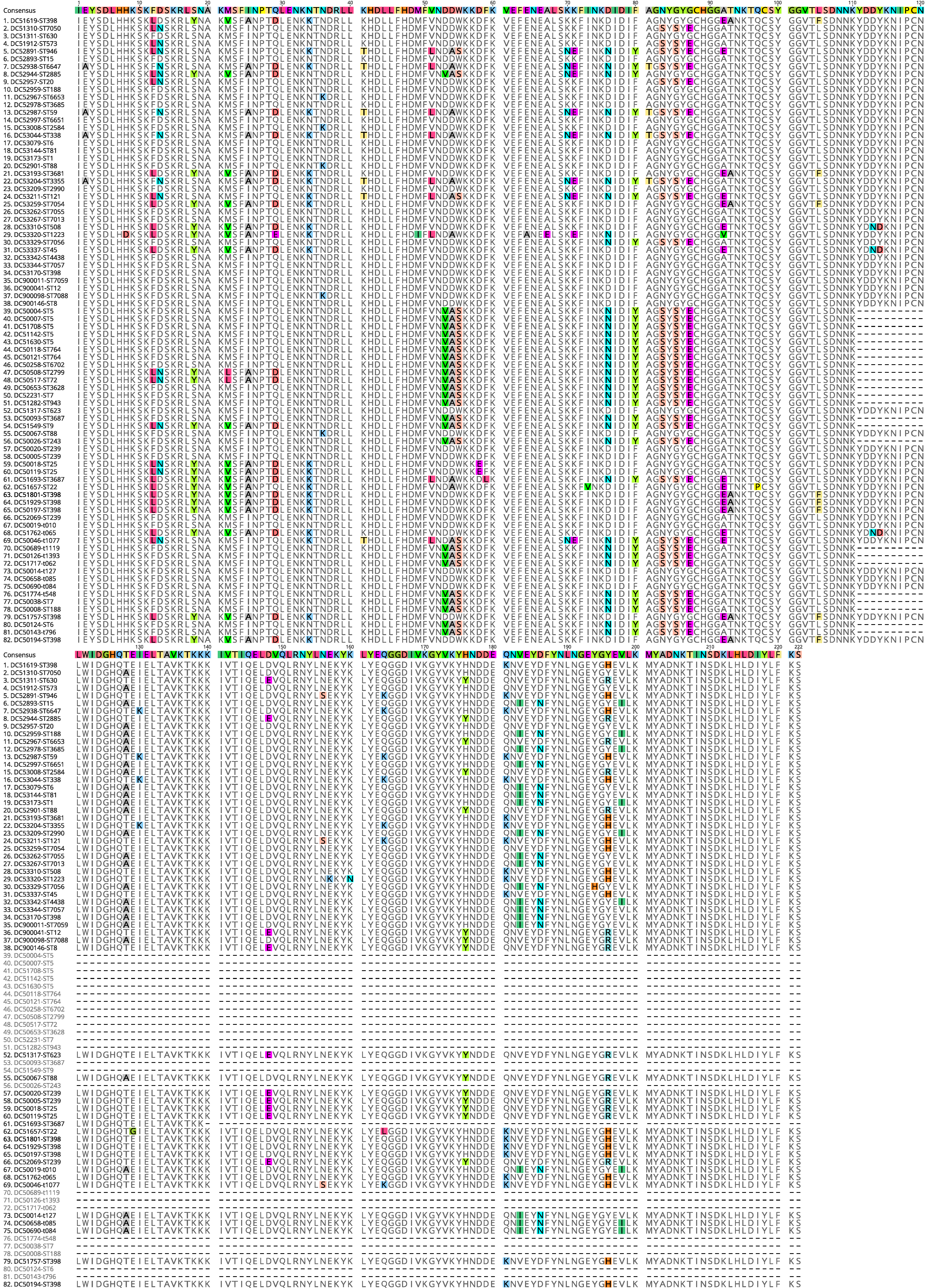

Supplement: Supplementary Figure S1.tif [file KVIR_A_2550622_SM9385.tif]

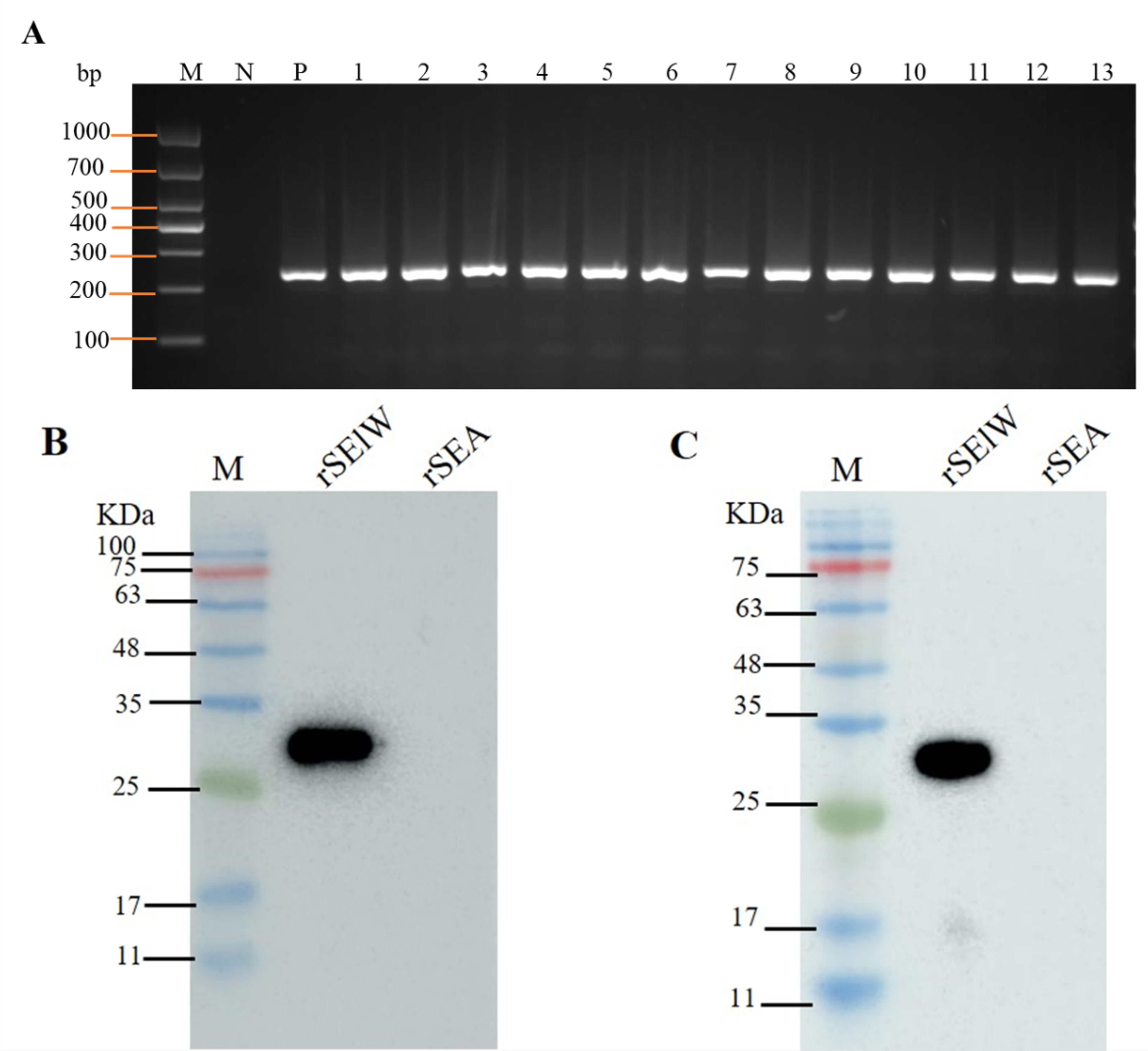

Supplement: Supplementary Figure S2.tif [file KVIR_A_2550622_SM9384.tif]
