# Supplementary material for: Preliminary assessment of the therapeutic potential of staphylococcal enterotoxin-like W via biological activity and TCR binding sites analysis
Source: Virulence. 2025 Aug 31;16(1):2550622. doi: 10.1080/21505594.2025.2550622 (PMC12407819; doi:10.1080/21505594.2025.2550622)
Supplement: Supplementary Table S1.docx [file KVIR_A_2550622_SM9388.docx]

Supplementary Table S1. Primers used in this study

| **Primer name** | **Amino acid substitution ^a^** | **Primer sequences ^b^** | **Using of primers** |
| --- | --- | --- | --- |
| rSElW |  | F: 5'-ccccggatccatcgaatattcagacttacatc-3' | Clonal expression of rSElW |
|  |  | R: 5'-ccccaagcttttatgatttgaataaatagatatct-3' |  |
| *selw* |  | Fw:5'-atgggggagaagccaataaaacgca-3' | *selw* gene detection |
|  |  | Rw:5'-tgtcgccaccttgttcgtacaactt-3' |  |
| Y18A | Tyr18→Ala | F18: 5-caagcgtcta***gct***aatgctaaggtgtcat-3' | Introduce the mutation site of Y18A |
|  |  | R18: 5-cttagcatt***agc***tagacgcttggaatcaag-3' |  |
| N19A | Asn19→Ala | F19: 5'-caagcgtctatat***gct***gctaaggtgtcat-3' | Introduce the mutation site of N19A |
|  |  | R19: 5'-tgacaccttagc***agc***atatagacgcttgg-3' |  |
| W55A | Trp55→Ala | F55: 5'-gtaaatgatgat***gcg***aaaaaagattttaa-3' | Introduce the mutation site of W55A |
|  |  | R55: 5-aaatctttttt***cgc***atcatcatttacgaac-3' |  |
| C88A | Cys88→Ala | F88: 5'-ggatacgga***gct***catgggggagaag-3' | Introduce the mutation site of C88A |
|  |  | R88: 5'-cccatg***agc***tccgtatccataatttcca-3' |  |
| C98A | Cys98→Ala | F98: 5'-aacgcaa***gct***agttatggtggtgttacttt-3' | Introduce the mutation site of C98A |
|  |  | R98: 5'-caccataact***agc***ttgcgttttattggctt-3' |  |

^a^ Specific amino acid substitution.

^b^ F and R refer to respectively forward and reverse primer. Primers Fw/Rw are reverse transcription primers for the *selw* gene. Restriction enzyme sites are underlined. The bolded letters are the mutated nucleotide residues.
