# Supplementary material for: Preliminary assessment of the therapeutic potential of staphylococcal enterotoxin-like W via biological activity and TCR binding sites analysis
Source: Virulence. 2025 Aug 31;16(1):2550622. doi: 10.1080/21505594.2025.2550622 (PMC12407819; doi:10.1080/21505594.2025.2550622)
Supplement: Clean copy of Supplementary Table S2- QVIR-2025-0062.R1.docx [file KVIR_A_2550622_SM9387.docx]

Supplementary Table S2. Strains tested in this study.

| **Number** | **Strains** | **Province** | **Source** | **Data** | **MSSA-MRSA** | **CC** | **MLST** | ***spa*** |
| --- | --- | --- | --- | --- | --- | --- | --- | --- |
| 1 | DC52069 | Hei Longjiang | patient | 2017 |  | CC8 | ST239 | t030 |
| 2 | DC52961 | Yun Nan | food | 2017 | MSSA | CC398 | ST6648 | t034 |
| 3 | DC51329 | Guang Dong | food | 2008 | MSSA | CC188 | ST188 | t189 |
| 4 | DC51630 | Hei Longjiang | patient | 2016 |  | C5 | ST5 | t002 |
| 5 | DC51757 | Hei Longjiang | patient | 2016 |  | CC398 | ST398 | t034 |
| 6 | DC50194 | An Hui | patient | 2005 |  | CC398 | ST3332 | t034 |
| 7 | DC50019 | Zhe Jiang | patient | 2009 |  | NA | NA | t010 |
| 8 | DC51911 | Hai Nan | food | 2006 | MSSA | CC398 | ST3332 | t034 |
| 9 | DC51326 | Guang Dong | food | 2007 | MSSA | C15 | ST15 | t084 |
| 10 | DC50005 | Zhe Jiang | patient | 2009 |  | CC8 | ST239 | t037 |
| 11 | DC51142 | Bei Jing | patient | 2005 |  | CC5 | ST5 | t002 |
| 12 | DC52931 | Yun Nan | food | 2016 | MRSA | CC9 | ST9 | t899 |
| 13 | DC51908 | Hai Nan | food | 2006 | MSSA | CC398 | ST398 | t588 |
| 14 | DC51619 | Shan Dong | patient | 2016 | MSSA | CC398 | ST398 | t571 |

NA: not analysis.
